# Biomedical Applications of CNT-Based Fibers

**DOI:** 10.3390/bios14030137

**Published:** 2024-03-07

**Authors:** Yun Ho Jeong, Mina Kwon, Sangsoo Shin, Jaegeun Lee, Ki Su Kim

**Affiliations:** 1 School of Chemical Engineering, Pusan National University, Busan 46241, Republic of Korea; dbsgh0919@gmail.com (Y.H.J.); kmn1126111@pusan.ac.kr (M.K.); shinsangsu15@gmail.com (S.S.); 2 Department of Organic Material Science and Engineering, Pusan National University, Busan 46241, Republic of Korea; 3 Institute of Advanced Organic Materials, Pusan National University, Busan 46241, Republic of Korea

**Keywords:** carbon nanotubes, fiber, biomedical, tissue engineering, biosensor

## Abstract

Carbon nanotubes (CNTs) have been regarded as emerging materials in various applications. However, the range of biomedical applications is limited due to the aggregation and potential toxicity of powder-type CNTs. To overcome these issues, techniques to assemble them into various macroscopic structures, such as one-dimensional fibers, two-dimensional films, and three-dimensional aerogels, have been developed. Among them, carbon nanotube fiber (CNTF) is a one-dimensional aggregate of CNTs, which can be used to solve the potential toxicity problem of individual CNTs. Furthermore, since it has unique properties due to the one-dimensional nature of CNTs, CNTF has beneficial potential for biomedical applications. This review summarizes the biomedical applications using CNTF, such as the detection of biomolecules or signals for biosensors, strain sensors for wearable healthcare devices, and tissue engineering for regenerating human tissues. In addition, by considering the challenges and perspectives of CNTF for biomedical applications, the feasibility of CNTF in biomedical applications is discussed.

## 1. Introduction

Carbon nanotubes (CNTs) are tubular-shaped carbon materials with nanoscale diameter. They have been considered representative nanomaterials owing to their remarkable mechanical and electrical properties [1]. Recently, the CNT market has been growing unprecedently fast with the rise of the battery industry; CNTs are replacing carbon blacks as conducting additives in batteries [2]. Keeping pace with the explosively increasing demand, the production capability also increases rapidly as the CNT synthesis technique improves, especially by fluidized bed reactors. However, the application of CNTs is still limited to batteries.

To explore new markets, other application areas of CNTs have been extensively investigated. Among various areas, biomedical applications are gaining special interest because they can create remarkable added value [3]. Due to the unique physical, chemical, and mechanical properties of CNTs, they have been utilized for various biomedical applications such as drug delivery carriers [4,5,6], tissue engineering scaffolds [7,8,9], biosensors [10,11], and imaging agents [12]. However, there are also challenges related to their biocompatibility, toxicity, and potential long-term effects [13]. To resolve these issues, techniques to assemble them into various macroscopic structures such as one-dimensional fibers, two-dimensional films, and three-dimensional aerogels have been developed. These macroscopic structures of CNTs extend the application area. Among these, CNT fibers (CNTFs) are gaining the biggest attention as they benefit the most from CNT’s one-dimensional characteristic [14,15,16]. Typically, the diameter of CNTFs ranges from several micrometers to tens of micrometers. CNTFs exhibit excellent mechanical and electrical properties in the macroscale world as their components, CNTs, do in the nanoscale. Taking advantage of fibrous structure, CNTFs open up new applications such as conductive wires, fiber-type supercapacitors and batteries, and reinforcement fiber in composites [17,18,19].

**Figure 1:**
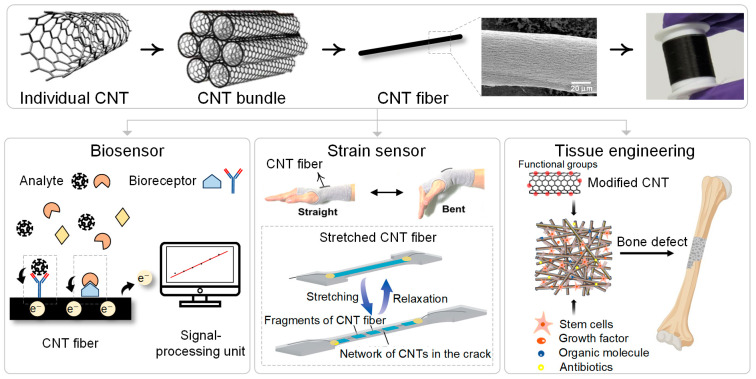
CNT nanofibers for various biomedical applications (reprinted with permission from the study by Tsentalovich et al. [16], reprinted with permission from the study by Liu et al. [23], reprinted with permission from the study by Zhou et al. [24]).

CNTFs are often compared with carbon fibers (CFs) because both have excellent mechanical strength. As they consist of carbon, which is very light, their specific strength is incomparably superb. Nevertheless, they also have very different properties. The uniqueness of CNTFs as compared to conventional CFs lies in their flexibility and conductivity. The electrical conductivity of the state-of-the-art CNTFs ranges from 1 to 10 MS/m while that of typical CFs ranges from 0.1 to 1 MS/m [20]. The flexibility is revealed by the knot efficiency. The knot efficiency is the ratio of the strength of a knotted fiber to that of an unknotted fiber. The knot efficiency of CNTFs is approximately 50 to 100 percent while that of CFs is only a few percent [21,22]. This high knot efficiency of CNTFs originates from their yarn-like structure [21]. Combining these unique properties of CNTFs, it is expected that CNTFs pioneer new applications that were not possible with conventional CFs. A natural insight suggests that biomedical application is a promising application area where CNTFs can fully exert their unique properties since it requires electrically conductive, mechanically robust, yet soft materials, as illustrated in Figure 1.

In this review, we introduce the recent research progress on the biomedical applications of CNTFs. First, we introduce CNTFs with a brief history of three representative spinning methods. Then, we introduce how CNTFs have been used in three areas of biomedical applications. CNTFs are used as biosensors to detect various analytes such as glucose, dopamine, and ascorbic acid, used as strain sensors to monitor human motion, and used in tissue regeneration. Finally, we provide our insights into future research directions for the biomedical applications of CNTFs.

## 2. Carbon Nanotube Fibers

CNTFs are one-dimensional macroscopic assemblies of CNTs. About twenty years ago, three techniques to assemble CNTs into a macroscopic CNTF were independently developed; they are called forest spinning, direct spinning, and wet spinning. Although the fiber formation mechanisms are different, in all three techniques, once CNTs are assembled into a fiber, they all maintain the structural integrity due to the strong van der Waals attraction force between CNTs and the resulting fibers being similar.

Forest spinning was first reported by Fan et al. in 2002 [25]. A vertically aligned CNT array grown on a substrate is often called a CNT forest. When the alignment of CNTs in a forest is very high (superaligned), it is possible to spin CNT fiber by pulling a part of CNTs from the CNT forest [26,27]. The key to this technique is to synthesize spinnable CNT forests. Forest spinning has some merits in academic aspects, so this technique has been widely investigated. Since we can measure and control the height of CNT forests, we can produce CNT fibers using CNTs of pre-defined length. In addition, the purity is high. However, it is not suitable for mass production due to the limited productivity.

The wet spinning of CNTF is similar to the conventional wet spinning of polymers from polymer solution. Preparing the high-quality dispersion of CNTs is the key to this technique. The first report on wet spinning used surfactant to disperse CNTs [28]. Later, it was reported that superacids can spontaneously dissolve CNTs forming charge-transfer complexes [29]. Using superacids, it became possible to make a high-concentration liquid–crystalline CNT dispersion, which dramatically improved the quality of CNTFs obtained from wet spinning [14,30,31,32]. The CNTFs from wet spinning have high packing density, so the mechanical and electrical properties are excellent. The limitation of this method is that it is difficult to obtain CNTFs consisting of long CNTs because of the difficulty in dispersing long CNTs.

Direct spinning is a method to spin CNTFs directly from the CVD reactor during the synthesis of CNTs by floating catalyst CVD. It is a simple one-step continuous process and does not require the dispersion of CNTs. Hence, the CNTs in the directly-spun CNTFs are typically longer than those from wet spinning. However, the directly-spun CNTFs have low packing density because the CNTs are not efficiently assembled during the spinning process. To overcome such limitations, Lee et al. proposed a combined strategy. They densified the directly-spun CNTFs using a superacid, which significantly improved the mechanical and electrical properties; the specific tensile strength and specific electrical conductivity were 4.08 N tex^−1^ and 2270 S m^2^ kg^−1^, respectively [22]. Wet spinning and direct spinning are still competing techniques and each technique does not have an absolute advantage. Depending on the purpose of the applications, one needs to choose a proper method to obtain CNTFs.

## 3. CNT Fibers-Based Biosensors

### 3.1. Basic Principles of Biosensors

A biosensor is a device that uses specific biochemical reactions mediated by isolated enzymes, immunosystems, tissues, organelles, or whole cells, to detect biological analytes usually by electrical, thermal, or optical signals [33]. Since a biosensor detects biological signals from the human body or environment, it can be used in broad applications including food [34], healthcare [35], medicine [36], environmental monitoring [37], and industrial testing [38]. Biosensors are required to be selective, sensitive, simple, rapid, cost-effective, and portable [39,40].

**Figure 2:**
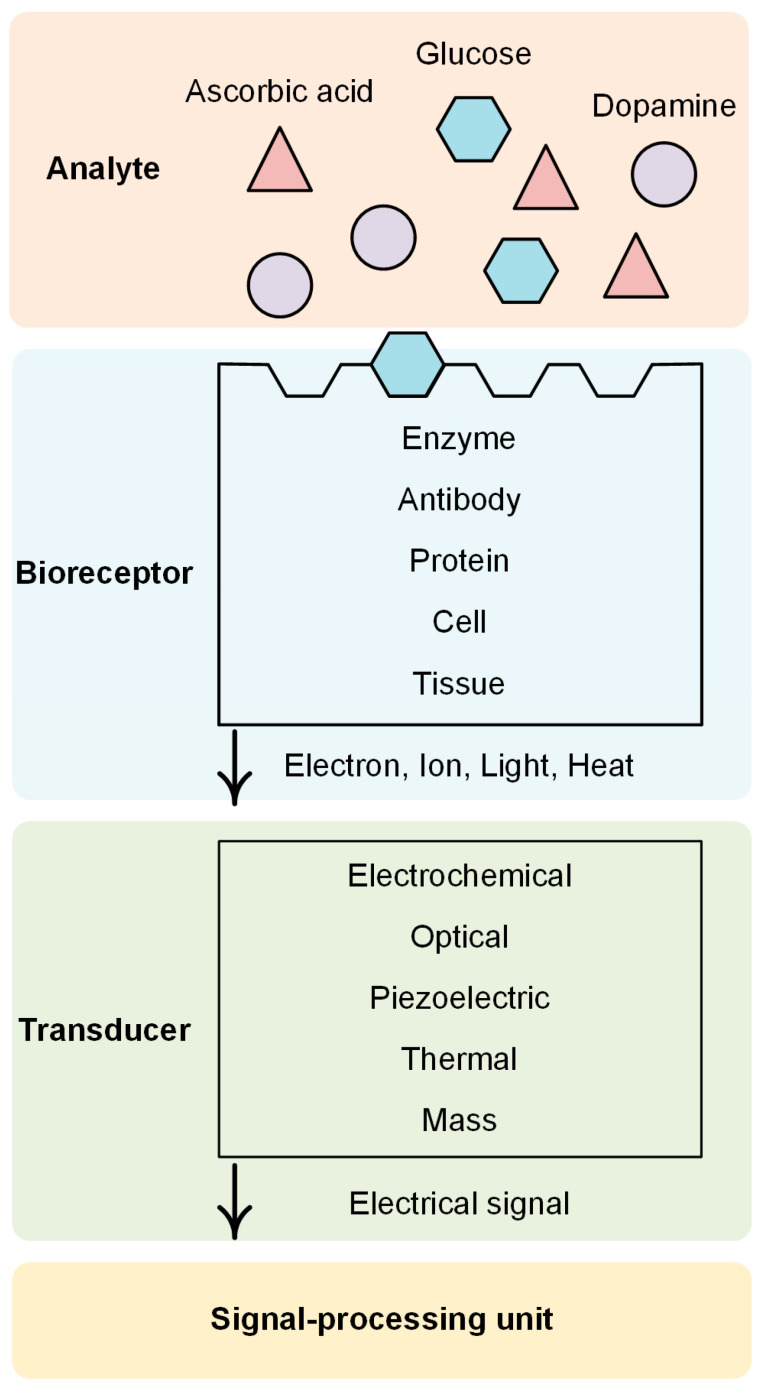
Biosensor components (bioreceptor, transducer, and signal-processing unit) and detection mechanism.

A typical biosensor consists of three components: a bioreceptor, a transducer, and a signal-processing unit (Figure 2). Bioreceptors are materials that sensitively interact with biological analytes such as antigens, glucose, neurotransmitters, toxins, etc. Examples of bioreceptors include enzymes, antibodies, proteins, nucleic acids, cells, and tissues. The interaction between a bioreceptor and an analyte produces signals in various forms such as electrons, ions, light, heat, and gases [41]. Transducers convert these signals into an electrical signal. Finally, the electrical signal is processed by a signal processing unit so that we can interpret the information of the analyte [42].

Among various types of transducers, electrochemical transducers are the most widely used ones. Electrochemical transducers are electrodes that obtain electrical signals from a chemical reaction and are subdivided into amperometric, potentiometric, and conductometric types. Amperometric sensors measure the varying current signals caused by the chemical reactions between analytes and bioreceptors at a constant potential. Potentiometric sensors measure the electric potential difference between working and reference electrodes with no current flow. Conductometric sensors measure the change of electrical conductivity or conductance caused by the change in the concentration of ionic species [43]. Electrochemical transducers have several advantages such as low cost, simple design, suitability for miniaturization, fast response, and high sensitivity [44,45]. In this section, we review various examples of CNTFs as effective transducers in biosensors.

### 3.2. CNT Fiber-Based Biosensors

Carbon materials have been widely used as electrodes in various applications due to their broad potential window, low cost, structural polymorphism, and chemical stability [46,47]. In addition, they offer rich electrochemical reaction sites due to their large surface area and abundant oxygen-containing functional groups at the surface [46]. Hence, carbon materials are suitable for electrochemical transducers in biosensors.

Among various carbon materials, carbon fibers (CFs) have been extensively investigated as microelectrode materials. They have several merits including small diameter (several micrometers), subsecond temporal resolution [48], less tissue damage [49], good electrochemical activity [50], biocompatibility [51], and high sensitivity for neurotransmitters detection [52,53]. However, carbon fiber microelectrode (CFME) has some limitations. For example, the CFME for amperometry measurement is not selective for analytes in complex environments such as a brain [54]. Moreover, the oxidation product of dopamine, which is highly reactive, could form an insulating film on the surface of CFME, which can degrade the measurement quality [55,56]. In addition, high impedance and low charge injection of CF limit its long-term use [57].

CNTs, one-dimensional tubular structures of the sp2 carbon network, are some of the most promising electrode materials for electrochemical sensing applications. CNT-based biosensors have demonstrated electroactivities superior to traditional electrodes such as Pt, Au, and glassy carbon electrodes, showing a shorter response time, high selectivity, high sensitivity, and low detection limit [58,59,60]. These advantages are attributed to their high electrical conductivity, high electron transfer rate, high surface area for the effective adsorption of biomolecules, low overvoltage, nanoscale size, and abundant redox active sites (edges of CNT) [61,62,63,64].

Taking advantage of these merits of CNTs, a CNT-modified CFME was developed by coating CNTs on the surface of CFs. CNT-modified CFMEs showed an improved electrochemical performance. However, there are some limits such as CNT aggregation on the electrode surface [65], low reproducibility, and alignments when CNT-modified CFME was fabricated via dip coating [66]. Moreover, there have been some concerns about CNT toxicity. For example, the cytotoxicity of CNTs was reported in alveolar macrophages [67]. In addition, inhalation and intravenous injection of CNTs cause immunotoxicity such as genotoxicity, oxidative stress, and inflammatory response because they interact directly with immune cells due to their nanoscale dimension [68,69,70].

The toxicity of CNTs can be circumvented by using a macroscopic assembly of CNTs. For instance, CNT films, pastes, coatings, composites, and fibers have been used as electrodes [71]. Especially, CNTFs, one-dimensional macroscopic assemblies of CNTs, enable macroscale application while preserving excellent anisotropic properties of individual CNTs as well as having a high surface area and remarkable electrocatalytic properties [71]. The high surface area and nanoporosity of CNTF enable efficient adsorption of bioreceptors and improve electrochemical properties because redox reactions are catalyzed at bioreceptors, which are adsorbed at the interface of the electrodes [72].

Unlike individual CNTs, it has recently reported that CNTFs are biocompatible. They showed no cytotoxicity in cell lines such as HEK-293 and SH-SY5Y, no immune response with blood, and no evidence of long-term toxicity induced by potential leachates of CNTFs [73]. Moreover, CNTF-based microelectrodes (CNTFMEs) are directly made of CNTF in a similar way to CFME. This simplifies the fabrication process and enhances reproducibility. In addition, CNTFMEs overcome the shortcomings of CNT-modified CFME with improved CNT alignment and avoid aggregation of CNTs coated on the electrode [74]. CNTFMEs also have several advantages such as better stability, flexibility, sensitivity, fouling resistance, and lower detection limit compared to CFMEs. The next section reviews the demonstration of these superior properties of CNTFMEs.

### 3.3. CNTF-Based Biosensors for Detecting Various Analytes

In the previous section, we examined the advantages and feasibilities of CNTF-based biosensors. Due to the remarkable electrical conductivity of CNTFs, they are used to detect various analytes such as glucose, dopamine, ascorbic acid (AA), etc. Moreover, the high surface area arising from the highly porous morphology of CNTFs enables biomolecules to adsorb on the surface of the CNTFs. Due to these advantages, the performance of CNTFMEs is superior to conventional electrodes. In this section, various applications of CNTF-based biosensors are reviewed.

CNTF-based biosensors have not been studied very actively and are still considered to be in the early stages. Wang et al. demonstrated the first CNTFMEs as biosensors, showing a better electrocatalytic response to important biomolecules, such as nicotinamide adenine dinucleotide (NAD), hydrogen peroxide, and dopamine, than CFMEs [75]. The results imply that CNTFME can be further utilized to detect much broader types of biomolecules with appropriate bioreceptors such as enzymes.

The type and spinning method of CNTFs used for the detection of various analytes are summarized in Table 1. Various analytes have been detected by CNTF biosensors including glucose, dopamine, AA, and so on. The CNTFs used in the CNTF-based biosensor were made by wet spinning, direct spinning, forest spinning, etc. These CNTFs include pure CNTF and composite fibers of CNTs and other materials.

#### 3.3.1. Glucose

According to the World Health Organization (WHO), the number of people suffering from diabetes has increased from 108 million in 1980 to 422 million in 2014. Diabetes can lead to disabilities such as the failure of eyes, nerves, kidneys, hearts, and various organs as well as death in severe cases [96]. Diabetics should periodically collect blood to check blood sugar levels and keep the glucose level constant. However, pricking one’s finger with a needle is a cumbersome process. For this reason, implantable glucose biosensors are attractive for continuous monitoring.

Implantable glucose biosensors are mostly amperometric enzymatic biosensors. A typical class of enzymes used for such enzymatic biosensors is glucose dehydrogenases (GDHs). GDHs are enzymes that catalyze the oxidation of glucose into D-glucono-δ-lactone in the presence of cofactors such as NAD, nicotine adenine dinucleotide phosphate (NADP), pyrroloquinoline quinone (PQQ), and flavin adenine dinucleotide (FAD) [97]. Among these various GDHs, NAD-dependent GDH shows better glucose specificity than the others [98]. NAD+ plays a role as an electron acceptor in the oxidation of glucose (1) [99]. However, the oxidation of NADH requires high applied potential due to its low electron transfer kinetics (2). Therefore, there have been attempts to decrease the overpotential by using redox mediators. These mediators enable fast and reversible NADH recycling, and thus fast glucose detection [100].

(1) $$Glucose+{NAD}^{+}\underset{Glucose\;dehydrogenase,(GDH)}{\to }D-glucono-\delta -lactone+NADH$$

(2) $$NADH\to {NAD}^{+}+{2H}^{+}+{2e}^{-}$$

Another famous enzyme for the detection of glucose is glucose oxidase (GOx). In fact, GOx is the most frequently used for the determination of glucose levels due to its high sensitivity and selectivity as well as low cost [101]. Enzymatic electrochemical glucose biosensors that use GOx can be subdivided into three generations depending on the method of measurement. The first generation is based on the measurement of oxygen consumption or generation of hydrogen peroxide during enzymatic reaction. The second generation is based on the measurement of electron mediators that shuttle electrons between the redox centers of enzymes and an electrode. The third generation is based on the measurement of direct electron transfer between the redox centers of enzymes and an electrode [102]. GOx catalyzes the oxidation of glucose into D-glucono-δ-lactone and hydrogen peroxide (3). The hydrogen peroxide is converted into oxygen, hydrogen, and electrons at a potential of about 700 mV (4). Then, the biosensor detects the concentration of glucose through the change of electrical current. Thus, the electron transport between the active site of GOx and the electrode surface is important [103]. In biological applications, the use of GOx is known to be suitable for an implantable sensor because biological environments have a large amount of coenzymes and oxygen [104].

The principle of detecting glucose using GOx is as follows [102]:

(3) $$Glucose+O_{2}\underset{Glucose\;oxidase,(GOx)}{\to }D-glucono-\delta -lactone+H_{2}O_{2}$$

(4) $$H_{2}O_{2}\to O_{2}+{2H}^{+}+{2e}^{-}$$

Viry et al. first demonstrated CNTFME, made solely of CNTs, that senses glucose by adsorption of a mediator on the surface of CNTFME [76]. In contrast to CFME, CNTFME allowed for various optimizations such as surface treatment and hot stretching. Surface treatment using a solution of polyoxometalate H_3_PMo_12_O_40_ (POM) in H_2_SO_4_ led to enhanced active surface area and made the mediator adsorb more easily. Hot stretching aligned CNTs, enhancing the electronic properties of the CNTFME. The combined effect of optimizations improved the electrochemical performance of CNTFME, enabling the CNTFME to outperform CFME in detecting glucose in terms of stability and sensitivity. The study showed the potential of new amperometric sensor devices.

Wang et al. reported that helical CNTF bundles that mimic the hierarchical structure of muscle can be used as a biosensor that detects various analytes including glucose [77]. These fibers provide stable fiber-tissue interaction since these fibers are flexible and have a low bending stiffness that matches that of tissues and cells. Due to their high surface area and excellent electrochemical properties, these fibers provide active sites for the immobilization of GOx. These CNTF electrodes showed a detection range of 2.5–7.0 mM, including, at the physiological level, a high sensitivity of about 5.6 nA μM^−1^ and a low detection limit of 50 μM. These CNTFs detected not only glucose but also ions, antigens, and hydrogen peroxide individually or simultaneously according to combining various active materials such as polymers, metals, metal oxides, and biomolecules.

Zhu et al. designed a novel brush-like CNTFME for glucose biosensors to maximize the surface area [71]. This electrode has a brush-like end that acts as electric flex and the individual nano-yarns within this brush-like end perform as multi-nano-electrodes that provide fast electron transfer and a high surface area of which enzymes can immobilize. GOx is immobilized at the end of the electrode, and the enzyme layer is encapsulated by a semi-permeable membrane that permeates glucose and oxygen. The generated electrons are captured by CNTF and signals are used to detect glucose concentration. This biosensor has superior sensitivity, detection range, and linearity for glucose detection compared to conventional Pt-Ir electrodes due to high electron mobility and it has been shown that the sensitivity can be increased through thermal annealing. The sensitivity of this biosensor increased from 0.96 nA mM^−1^ to 7.2 nA mM^−1^ by annealing. In addition, this electrode is suitable as an implantable glucose biosensor since it detects glucose of 2–30 mM, which is a physiological blood glucose level. They further developed their CNTFME design using a gold coating, which lowered the minimum detection concentration from 2 mM to 25 μM [78].

**Figure 3:**
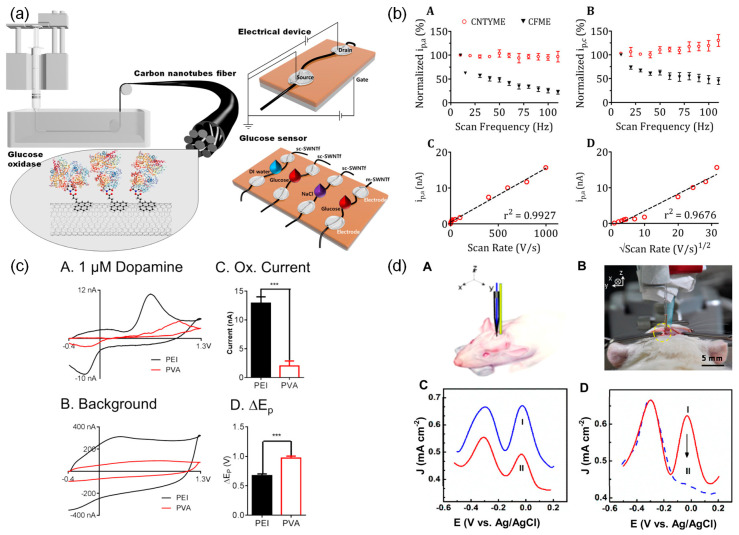
CNTF-based biosensors for various applications: (**a**) Schematic illustrations of glucose biosensors using CNTF with anchored glucose oxidase (reprinted with permission from the study by Lee et al. [79]), (**b**) Dopamine biosensor using CNTF compared to carbon fiber. (**A**) Peak oxidation current, and (**B**) peak reduction current at CNTFMEs (red circles) and CFMEs (black triangles). Oxidation current for a CNTFME for 1 μM dopamine plotted against (**C**) scan rate and (**D**) the square root of scan rate (reprinted with permission from the study by Jacobs et al. [54]). (**c**) Dopamine biosensor using CNTF with different polymers. (**A**) Cyclic voltammograms of 1 μM dopamine for PEI-CNT (black) and PVA-CNT (red) fiber electrodes. (**B**) Background charging current for the same electrodes. (**C**) Average peak oxidative currents for 1 μM dopamine. (n = 6 each, *** *p* < 0.0001, *t*-test, error bars SEM) (**D**) ΔE_P_ values of the electrodes (reprinted with permission from the study by Zestos et al. [74]). (**d**) Ascorbic acid biosensor using CNTF to measure ascorbic acid level in live rat brain. (**A**) Schematic illustration of the in vivo setup for determining AA in rat brain. (**B**) Optical images before and after the stereotaxic implant into the brain (yellow circle). (**C**) Differential pulse voltammetry (DPV) recorded at the CNTFME in the striatum of normal rat (I) and rat brain models of AD (II). (**D**) DPV responses recorded at the CNTFME in the striatum of the rat brain model of AD before (I) and after (II) injection of ascorbate oxidase. (reprinted with permission from the study by Zhang et al. [91]).

Lee et al. made a semiconducting SWCNT (sc-SWCNT) fibers-based glucose biosensor (Figure 3a) [79]. Individual sc-SWCNTs have been used as material for biosensors. In their work, they separated SWCNTs into sc-SWCNTs and metallic SWCNTs (m-SWCNTs). The separated SWCNTs were wet spun into fibers separately and an enzyme capable of reacting with glucose was immobilized on the fibers. When sensing glucose, the electrical current in sc-SWCNT fiber changed while m-SWCNT fiber did not. This is because sc-SWCNT fiber exhibited a field effect while m-SWCNT fiber did not [105]. In addition, sc-SWCNT showed a detection limit of 0.5 μM and good sensitivity in the presence of water (99%) and NaCl solution (0.4–1%), the main constituents of sweat. The study demonstrates the potential of sc-SWCNT fibers as a wearable glucose biosensor platform.

Not only fiber-type CNTFMEs but also membrane-type CNTFMEs have been developed by using electrospinning and solution blow spinning. Manesh et al. fabricated a glucose biosensor by immobilizing GOx on an electrospun nanofibrous electrode [80]. The nanofibrous electrode consists of polymethylmethacrylate (PMMA) and MWCNTs which are wrapped by a cationic polymer poly(diallyldimethylammonium chloride) (PDDA). Owing to the nanofibrous structure, the electrode has a large surface area. In addition, the MWCNTs wrapped by PDDA had a strong electrostatic interaction with GOx, which promoted the immobilization of GOx. This electrode showed high reusability, scalability, selectivity, stability, and reproducibility. Oliveira et al. reported another membrane-type glucose biosensor using a solution blow spun nanocomposite fibers that consist of poly(lactic acid) (PLA) and MWCNTs [81]. Solution blow spinning is a method of fiber spinning that offers high surface area and good productivity [106]. PLA is a material that is widely used in applications in biosensors, biomaterials, and filtration in the form of electrospun fibers and solution blow spun fibers. PLA/MWCNTs nanofibers function as good support for enzyme immobilization with high porosity. The biosensor manufactured at the optimized condition showed an excellent sensitivity and detection limit: 358 nA mM^−1^ and 0.08 mM, respectively. This work demonstrates that blow-spun nanocomposite fibers offer great potential for applications as amperometric biosensors. Feng et al. designed an electrode using CNT film fiber for a glucose biosensor [82]. The CNT thin film was spun directly from a CVD reactor. They showed that acid treatment improves the sensitivity from 0.153 μA mM^−1^ to 4.867 μA mM^−1^.

Enzymatic glucose sensors have some disadvantages such as the complex immobilization procedure as well as susceptibility to changes in pH, temperature, and humidity. To circumvent these disadvantages, enzyme-free glucose sensors are drawing attention. Muqaddas et al. made an enzyme-free CNTF-based glucose biosensor by depositing copper oxide (CuO) nanoparticles on the surface of CNTFs to improve the electrocatalytic activity of sensors for glucose detection [83]. This biosensor showed excellent performance in detecting glucose with a high sensitivity of 3.025 mA (cm^2^ mM)^−1^, a low detection limit of 1.4 μM, and a wide linear range of up to 13 mM. In recent research, the authors employed a composite of nickel and cobalt selenide (NiCo-Se) integrated into CNTFs to exploit its electrocatalytic properties for glucose sensing. The porous nature of the CNTF facilitated an increased provision of active sites, coupled with the enhanced electron transfer rate attributed to NiCo-Se, resulting in outstanding performance in glucose sensing. The sensor exhibited remarkable characteristics, including excellent sensitivity of 813 mA (cm^2^ mM)^−1^, a low detection limit of 0.59 μM, and a dynamic range extending up to 10 mM. Furthermore, the sensor maintained superior stability by achieving 92% of its original value over 50 cycles, demonstrating its robustness for practical applications [84].

#### 3.3.2. Dopamine

Dopamine is an important neurotransmitter associated with the cardiovascular, renal, and central nervous systems. Abnormal levels of dopamine can cause neurological diseases and disorders such as schizophrenia [107], depression [108], and Parkinson’s disease [109]. Since dopamine is an electrochemically active compound, it can be sensitively and selectively detected by electrochemical methods [110,111]. However, it is hard to detect dopamine accurately because the oxidation potential of dopamine is similar to that of AA whose concentration is almost three orders higher than that of dopamine [112]. Thus, accurate detection of dopamine is hard due to overlapped oxidation signals. The second reason is electrode fouling which degradezs the quality of measurement after dopamine oxidation [113]. There are some attempts to fabricate dopamine biosensors by using CNTFs to overcome these limitations.

Several reports demonstrated that CNT-modified electrodes improve electrochemical properties and lead to a shift of the oxidation potentials of dopamine and AA [110,111,114]. Viry et al. utilized these advantages for discrimination between dopamine and AA using CNTFME [85]. When using this electrode, the oxidation peak of dopamine is shifted and those of AA are also suppressed. In addition, this electrode showed between one and two orders of magnitude higher currents than conventional glassy carbon electrodes. This superior property is accounted for more efficient electron transfer caused by electrostatic effects, favorable π-π interaction between CNT and dopamine, and high surface area of CNTF.

For long-term use, the electrodes are required to have high resistance to dopamine fouling. Harreither et al. compared the resistance to dopamine fouling of CNTFMEs and CFMEs [86]. During dopamine oxidation at 100 μM, the current of CFMEs decreased by about 50%, whereas the current of CNTFMEs did not decrease. In addition, CNTFMEs are easy to handle since they have similar dimensions to traditional CFMEs and they don’t require any additional modification or preactivation. Wang et al. detected dopamine concentration for long-term in vivo for about 8 weeks by using CNTFs, which are flexible and possess a low limit of detection and a wide linear range [87]. Furthermore, this fiber showed good stability after hundreds of bending and remarkable compatibility with neurons after implantation.

Fast-scan cyclic voltammetry (FSCV) is a technique that can detect neurotransmitters in complex environments such as the brain in real-time, on a subsecond-to-second timescale [115]. This method also minimizes tissue damage by using micro-size probes and has a high sensitivity to detect dopamine at low concentrations ranging from nanomolar to micromolar. Schmidt et al. fabricated CNTFMEs and detected neurotransmitters including dopamine in living brain tissue by using FSCV [88]. They found that CNTFMEs had better detection of neurotransmitters than conventional CFMEs. CNTFMEs exhibited higher sensitivity and a lower limit of detection of 13.4 ± 1.7 nM than CFMEs of 20.8 ± 1.3 nM. Jacobs et al. also showed that CNTFMEs with FSCV had faster measurement rates compared to conventional CFMEs by about two orders of magnitude (Figure 3b) [54]. Oxidation of dopamine occurs on the surface of the microelectrodes, and the adsorption time for dopamine decreases when the frequency of FSCV scan repetition increases. CNTFMEs, which are more reversible in dopamine oxidation than CFMEs, enable high temporal measurement of neurotransmitter dopamine sensing (Figure 3(bA,bB)). The authors changed the scan rate to confirm whether the dopamine kinetic was a diffusion-limited process or an adsorption-limited process. They explained that dopamine kinetics is an adsorption-limited process because the oxidation current is linear to the scan rate rather than linear to the square root of the scan rate (Figure 3(bC,bD)) [116]. Schmidt et al. and Jacobs et al. attributed the superior sensitivity and temporal measurement of CNTFMEs to their higher electron transfer kinetics.

CNTFMEs can also be fabricated using CNTFs spun by a wet spinning process with a polymer [75,85,86]. CNTs are dispersed in an aqueous solution using a surfactant. Then the CNT dispersion is injected into a coagulation bath that contains poly(vinyl alcohol) (PVA) solution. The PVA adorbs onto the surface of CNTs and displaces surfactants to form a CNTF. These CNTFs are actually polymer/CNT composite fibers.

Usually, polymer/CNT composite fibers including PVA-CNTFs suffer from low electrical conductivity due to the low conductivity of the polymer [117]. Thus, there have been efforts to improve the electrical conductivity of polymer/CNT composite fibers. For example, using polyethyleneimine (PEI) as a coagulant instead of PVA improved the electrical conductivity by a hundred times due to the physisorption of the amine to the sidewall of CNTs. The amine groups of PEI bind CNTs and facilitate charge transfer with good conductive properties [118].

Zestos et al. improved the electrochemical properties of electrodes by using PEI-CNTFs, which are more conductive than PVA-CNTFs [74]. PEI-CNTFMEs showed higher sensitivity and a lower limit of detection of 4.7 ± 0.2 nM compared to PVA-CNTFMEs (53 ± 5 nM) and CFMEs (24 nM). Regardless of the presence or absence of dopamine, PEI-CNTFs show a larger capacitive current than PVA-CNTFs due to a larger electroactive surface area or greater surface roughness (Figure 3(cA–cC)). The potential difference between peaks is smaller for PEI-CNTFs, indicating that the electron transfer kinetics of PEI-CNTFs are faster than those of PVA-CNTFs. (Figure 3(cD)). In addition, these electrodes maintained a stable signal for dopamine detection with negligible loss compared to CFMEs. Yang et al. investigated the effect of surface properties of CNTFs, such as surface roughness and oxygen content, on the electrochemical performance of dopamine detection. They fabricated and compared three different CNTFMEs using PEI-CNTFs, chlorosulfonic acid (CSA)-CNTFs, and forest-spun CNTFs [89]. Among them, forest-spun CNTFMEs had the highest sensitivity for dopamine. This was attributed to the abundant oxygen-containing functional groups on the surface of forest-spun CNTFs, which are negatively charged at physiological pH and interact electrostatically with positively charged dopamine molecules [119]. In addition, small crevices on the fiber surface trapped dopamine during FSCV detection, making the current independent of scan frequency. Similarly, the authors conducted laser treatment on CNTFME to increase its surface area and introduce oxygen functional groups. This modification provided more adsorption sites for dopamine on the electrode, resulting in superior sensitivity and a lower limit of detection. The enhanced electrochemical performance observed in this study highlights the potential of surface-modified CNTFME for improved dopamine sensing applications [90]. Therefore, CNTFs with high electrical conductivity, small crevices, and abundant oxygen-containing functional groups are advantages for dopamine sensing.

#### 3.3.3. Ascorbic Acid

AA (Vitamin C) plays an important role as an antioxidant and free radical scavenger which protects tissues from oxidative stress [120]. It also participates in tissue growth, wound healing, metabolism, and collagen formation. Deficiency of AA can cause anemia, scurvy, infections [121] as well as neurodegenerative diseases such as ischemia [122], Parkinson’s disease [123], and Alzheimer’s disease (AD) [124]. Since AA is an electroactive material such as dopamine, it can be easily oxidized and can be detected by electrochemical methods [125].

Zhang et al. developed CNTFMEs to measure AA levels in live rat brains with AD (Figure 3(dA,dB)) [91]. These electrodes achieved high selectivity and sensitivity for AA detection by engineering defects and oxygen-containing groups in CNTFs. As shown in Figure 3(dC), two distinct peaks were observed at around −290 mV and −60 mV in normal rat brain (Curve 1) and in a rat brain model of AD (Curve 2), whereas only one peak was observed at around −290 mV in pure artificial cerebrospinal fluid, indicating the presence of AA. Moreover, the intensity of the peak decreased in a rat brain model of AD (Figure 3(dD)). Interestingly, the peak at −290 mV remained unchanged after injecting AAox, an enzyme that activates the redox reaction of AA and reduces the level of AA, suggesting that this electrode offers an effective internal reference for accurate detection in complex brain environments. This electrode possessed anti-fouling performance, high scanning stability compared to CFMEs, and good reproducibility.

Wang et al. fabricated an AA sensor using ferrocene methanol (Fc-OH) modified CNTF. The resulting Fc-OH/CNTF sensor demonstrated exceptional flexibility, high stretchability, and excellent bendability, along with notable electrocatalytic activity for AA oxidation. The fabricated sensor exhibited a wide linear range (3 μM to 3.0 mM) for AA, a low detection limit of 1.32 μM, a prolonged lifetime, and a rapid response speed (2.83 s). Notably, even after 100 and 500 bending cycles, the Fc-OH/CNTF sensor retained 90% and 60% of its initial activity [92].

#### 3.3.4. Oxygen and pH

Neuronal activity and energy metabolism in the brain are highly dependent on constant oxygen delivery. However, oxygen levels can also cause problems for the brain. Excessive levels of oxygen cause oxidative stress, which damages neurons and contributes to disorders such as epilepsy, schizophrenia, Parkinson’s disease, and Alzheimer’s disease. On the other hand, deficiency of oxygen during intense hypoxia and ischemia leads to energy failure and subsequent neuronal [126,127]. Another factor that affects brain function is pH. Brain pH is related to epileptic activity, and pH control can be a therapeutic strategy [128]. Furthermore, extracellular pH influences many physiological activities such as ion transmission, enzymatic activity, immune system, blood flow, etc. [129,130]. Dysregulated pH is closely related to cancer growth [131]. Thus, it is important to monitor the levels of oxygen and pH in the live brain for disease prevention and brain function.

Liu et al. developed a biosensor that can simultaneously detect the level of oxygen and pH in the brain. The sensor uses Hemin-Fc, synthesized by connecting a hemin group to two aminoferrocene through an amide bond [93]. This material was immobilized on the surface of forest-spun MWCNTFs. As the oxygen concentration increases, the reduction current peak increases. Conversely, as the pH decreases, the reduction current peak potential positively shifts. This suggests that this sensor can detect both oxygen and pH at the same time. Additionally, this biosensor showed high spatial and temporal resolution, long-term stability, and high selectivity.

#### 3.3.5. Malaria Biomarker (PfHRP2)

According to the World Health Organization (WHO), there have been 1.5 billion malaria cases and 7.6 million malaria-related deaths since 2000. In 2019 alone, 229 million people suffered from malaria infections and 409,000 people died from malaria [132]. Malaria is caused by four different types of human malaria protozoan species: Plasmodium falciparum, Plasmodium vivax, Plasmodium malariae, and Plasmodium ovale. Of these, Plasmodium falciparum (*P. falciparum*) is the most lethal to humans, causing serious disease and death [133]. *P. falciparum* synthesizes several kinds of proteins known as *P. falciparum* histidine-rich proteins (PfHRPs). One of these proteins, PfHRP2, shows high density and is more sensitive and selective compared to other proteins. Thus, there have been several attempts to detect this protein to diagnose malaria using techniques such as microscopy, flow cytometry, mass spectrophotometry, microarrays, and polymerase chain reaction (PCR). However, these methods are time-consuming, costly, and have low sensitivity [94,134]. To overcome these shortcomings, electrochemical methods are widely used. These electrochemical biosensors detect changes in electrical resistance as a result of interaction between analytes and the biosensor.

Paul et al. developed a chemoreceptive biosensor for the detection of malaria biomarker PfHRP2 using MWCNT-zinc oxide (ZnO) electrospun-nanofiber [94]. The MWCNT-ZnO nanofibers were obtained by electrospinning, a simple and low-cost method, followed by calcination, which created carboxylic groups (–COOH) on the nanofiber surface. Next, the nanofiber mat is deposited on a substrate and carboxylic groups (–COOH) are activated on the surface of the nanofiber mat by an activator and a coupling agent. Anti-HRP2 antibodies were then immobilized on the nanofiber surface by forming strong amide bonds. Finally, this biosensor detects the change in the electrical resistance of the nanofiber caused by the interaction between anti-HRP2 and HRP2. Since this device is flexible, cost-effective, and simple, it is advantageous for point-of-care (POC) diagnosis. Furthermore, it also has a wide detection range of 10 fg mL^−1^–10 ng mL^−1^, good sensitivity of 8.29 kΩg^−1^ mL, low detection limit of 0.97 fg mL^−1^, reproducibility, and specificity.

#### 3.3.6. Catechol

Catechol, a phenolic compound, is considered an environmental pollutant by the US Environmental Protection Agency (EPA) and the European Union (EU) due to its toxicity and difficulty in degrading the environment, even at low concentrations [135]. Skin contact with catechol can cause eczema and large amounts can induce depression of the central nervous system in animals [136]. In addition, it was reported that catechol reduces glutathione levels and causes cell death mainly by apoptosis [137]. Therefore, the determination of catechol is very important to protect the environment and human health. Several analytical methods for catechol detection have been developed such as liquid chromatography, capillary electrochromatography, spectrophotometry, and electrochemical methods [138]. Among these methods, biosensors based on immobilized peroxidases, such as horseradish peroxidase [139], laccase [140], and polyphenol oxidase (PPO) [141] have been used for catechol detection because of their cost-effectiveness, fast response time, high sensitivity, and low detection limit [142]. The principle of measuring phenolic compounds using electrodes with immobilized peroxidase is based on the fact that the reduction current of quinones or radicals caused by enzymatic oxidation of phenol in the presence of hydrogen peroxide is proportional to the phenol concentration [143].

Bourourou et al. developed a biosensor based on polyacrylonitrile (PAN)-CNT nanofibers to detect catechol by immobilizing PPO on the electrode surface [95]. The nitrile groups of these fibers were reduced to the amine groups, which served as anchoring points for PPO immobilization. They compared the performance of the reduced PAN-CNT nanofiber electrodes for different times, 1 h and 4 h. The high sensitivity of PAN-CNT nanofibers reduced for 4 h is attributed to the improvement in the diffusion of o-quinone and/or an increase in the electroactive surface of fibers. This electrode showed excellent catechol detection with a sensitivity of 118 mA M^−1^, a maximum current of 10.66 μA, and a detection limit of 0.9 μM.

## 4. Flexible Strain Sensors

A flexible strain sensor is a flexible device that converts applied mechanical strain into an electrical signal to detect various human motions. It can be either embedded in clothing or attached directly to the body. A good flexible strain sensor requires various properties such as stretchability, sensitivity, repeatability, durability, and linearity between strain and resistance change. The sensitivity of a strain sensor is represented by the gauge factor, which is defined as the ratio of the relative change in electrical resistance (R) to the mechanical strain (ε).

(5) $$Gauge\;factor=\left(∆R/R_{0}\right)/\varepsilon$$

**Figure 4:**
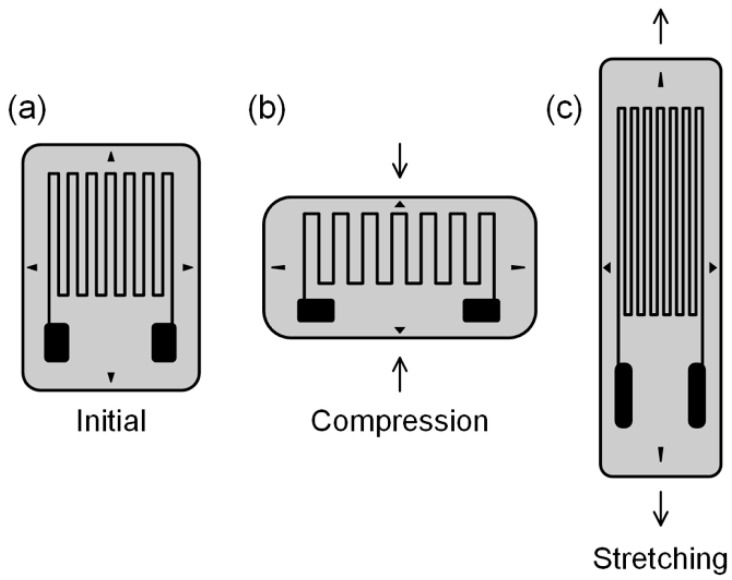
A traditional strain sensor consisting of an insulating flexible substrate that supports a metallic foil pattern. Deformation of the sensor causes deformation of the metallic foil and a change in its electrical resistance. (**a**) initial state of the strain sensor, (**b**) compressed state, (**c**) stretched state.

A traditional type of strain sensor has an insulating flexible substrate that sustains a metallic foil pattern (Figure 4). As the strain sensor is strained, the metallic foil is also strained, causing a change in its electrical resistance. Such a change in electrical resistance under mechanical strain is termed piezoresistive effect. Traditional strain sensors that use metal as a piezoresistive material have low stretchability and sensitivity [144]. To overcome these limitations, composite strain sensors use composites made of piezoresistive materials and polymer as flexible substrates. Some examples of such composites are graphene/Ecoflex [145], graphene/poly(dimethylsiloxane) (PDMS) [146], graphene/poly(styrene-butadiene-styrene) (SBS) [147], rubber band/carbon black/PDMS [148], carbon black/silver nanoparticle/thermoplastic polyurethane (TPU) [149], nitrile butadiene rubber/carbon black/polydopamine [150], silver nanowire/PDMS [151], and ZnO nanowire/polystyrene [152].

### 4.1. CNTs/Polymer Composite Fiber Strain Sensors

Among various piezoresistive materials, CNTs have attracted attention because of their flexibility, high aspect ratio, and remarkable mechanical, electrical, and piezoresistive properties [153]. To take advantage of these properties, various CNTs/polymer composite strain sensors have been developed, where CNTs act as conductive fillers. The CNTs/polymer composite has a low percolation threshold for electrical conductivity due to the high aspect ratio of [154,155] addition, the tunneling effect among dispersed CNTs in polymer increases the electrical conductivity. Many of the previous studies reported film-shaped CNTs/polymer composite strain [156,157,158]. In this review, we limit our scope to fiber-shaped strain sensors.

**Figure 5:**
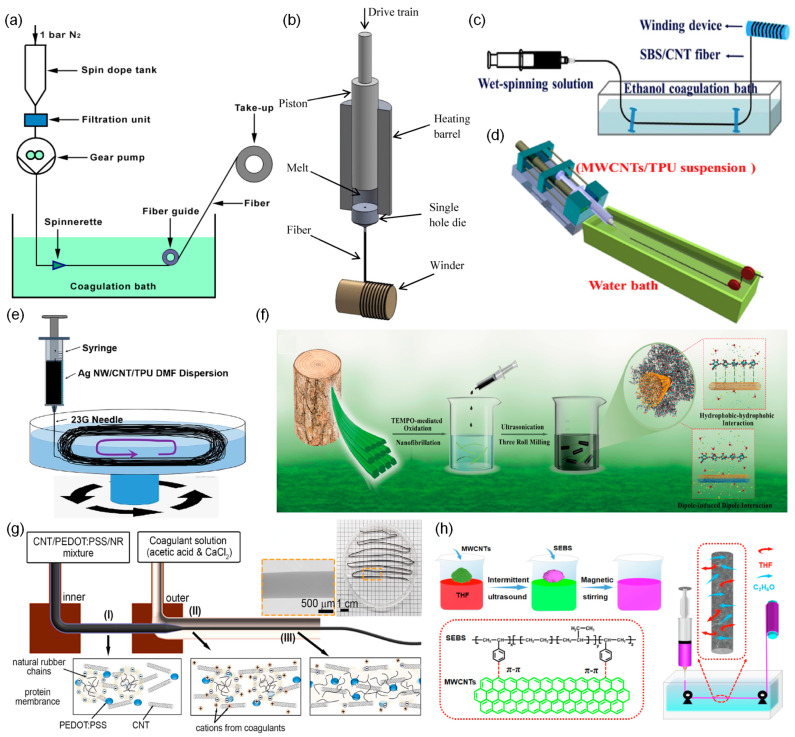
Schematic illustrations of the fabrication process of CNT/polymer composite fiber-shaped strain sensors. (**a**) MWCNTs/cellulose composite fiber (reprinted with permission from the study by Schulz et al. [159]), (**b**) MWCNTs/PC composite fiber (reprinted with permission from the study by Bautista-Quijano et al. [160]), (**c**) MWCNTs/SBS composite fiber (reprinted with permission from the study by Wang et al. [161]), (**d**) MWCNTs/TPU composite fiber (reprinted with permission from the study by Wang et al. [162]), (**e**) MWCNTs/silver nanowire/TPU composite fiber (reprinted with permission from the study by Zhang et al. [163]), (**f**) SWCNTs/cellulose nanofibrils composite fiber (reprinted with permission from the study by Wan et al. [164]), (**g**) MWCNTs/PEDOT:PSS/natural rubber composite fiber, step I: before injecting the mixture into coagulation solution, Step II: neutralization of natureal rubber chains in coagulation solution, step III: coagulation of neutralized natural rubber (reprinted with permission from the study by Lam et al. [165]), and (**h**) MWCNTs/SEBS composite fiber (reprinted with permission from the study by Li et al. [166]).

The development of lightweight and flexible fiber-shaped electronic devices and their integration into textiles is important for applications in healthcare, work wear, and sportswear. A notable example is the novel MWCNTs/cellulose composite fiber-shaped strain sensor developed by Qi et al. [159]. The sensor was produced through wet-spinning using an aqueous NaOH/urea solution as the solvent (Figure 5a). Cellulose served as the matrix material, chosen for its deformability, softness, washability, and durability. Remarkably, this sensor can be stretched by 14.3% strain and exhibits a gauge factor of 18 (Table 2).

Bautista-Quijano et al. fabricated a fiber-shaped strain sensor made of MWCNTs/polycarbonate (PC) composite using melt spinning (Figure 5b) [160]. They evaluated the mechanical, electrical, and strain-sensing properties of their sensor as a function of the MWCNTs’ weight concentration. The addition of a small amount of MWCNTs (less than 2 wt%) decreased the electrical resistivity of the fiber. Concentrations higher than 3 wt% resulted in residual MWCNT agglomerates and significantly reduced the sensor’s capability. The spinnability of MWCNTs/PC dispersion decreased at concentrations above 4 wt%. The electrical percolation threshold of the bulk material for undrawn-extruded rods was near 1 wt%, but that of the melt spun-fibers depended on the draw dawn ratio. Their sensor could be stretched by 9% and exhibited a maximum gauge factor of 16 at 3.5 wt%.

Yu et al. developed a fiber-shaped strain sensor made of MWCNTs/SBS composite using wet spinning (Figure 5c) [161]. They dispersed MWCNTs in tetrahydrofuran (THF) solvent and used SBS as a matrix due to its strong interfacial π–π interaction with MWCNTs. Remarkably, they achieved an impressive gauge factor of 20,000 under 50% deformation and a wide working range of 260%. One-dimensional fiber-shaped strain sensors offer an additional advantage: they can be seamlessly woven into everyday clothing or affixed to irregular surfaces.

Wang et al. made a fiber-shaped strain sensor made of MWCNTs/TPU composite via wet spinning (Figure 5d) [162]. They dispersed MWCNTs in dimethylformamide (DMF) solvent and chose TPU as the matrix due to its good flexibility and plasticity. The resulting composite fiber strain sensor exhibited remarkable properties: high stretchability (up to 320%), high sensitivity (gauge factor of 22.2 within a 160% strain and 97.1 within the range of 160–320%), and high durability (9700 cycles at a strain of 100%). These results highlight the potential of CNTs/polymer composite sensors for detecting human motions, including finger, elbow, and knee bending, as well as squatting and squat-jumping.

In a similar endeavor, He et al. fabricated a fiber-shaped strain sensor using a composite of MWCNTs and TPU through wet spinning. They meticulously investigated the sensor’s structural, mechanical, electrical, and strain-sensing properties as a function of the MWCNT content [167]. As the content of MWCNTs decreased, the pore size in the cross-section of the fiber increased. The sensor achieved high tensile strength (28 MPa) and conductivity (6.77 S/cm) with a MWCNTs to TPU weight ratio of 1:8. The strain at failure was as high as 565% when the weight ratio was 1:20. It showed a large gauge factor of 16,000 but a limited strain sensing range below 35%.

He et al. later improved the stretchability of this type of sensor [168]. They demonstrated a highly stretchable strain sensor with high sensitivity; the gauge factor was approximately 2800 in the strain range of 5–100%. They also demonstrated that their strain sensor could monitor the weight and shape of an object based on the 2D mapping of resistance changes, indicating good weavability and lightweight nature. They also developed a highly conductive MWCNTs/silver nanowire/TPU composite fiber-shaped strain sensor using wet spinning (Figure 5e) [163]. They used silver nanowires to increase conductivity. The effect of silver nanowire content on the mechanical, electrical, and strain-sensing performances of their fiber was investigated. The sensor had a tensile strength of 40 MPa and electrical conductivity of 0.803 S/cm. The addition of silver nanowire increased stretchability from 130 to 254% but decreased the gauge factor. The sensor could be freely written into various design patterns, making it useful in wearable smart textiles.

Wan et al. produced a multifunctional SWCNTs/cellulose nanofibrils composite fiber-shaped strain sensor using wet spinning (Figure 5f) [164]. The fiber was twisted and coated with polyvinyl alcohol (PVA) to improve mechanical performance and then adhered to a PE film. Cellulose nanofibrils served as the matrix material, aiming to mitigate SWCNT aggregation and stabilize the interfaces between cellulose nanofibrils and SWCNTs. The researchers employed a combination of a three-roll mill and ultrasonication to create a gel-like SWCNTs/cellulose nanofibrils dispersion in water. The sensor exhibited a strain range of 11.7% and an electrical conductivity of 86.43 S/cm.

Gue et al. introduced a strategy based on the intermolecular self-assembly of dopamine-conjugated carboxymethyl cellulose (DA-CMC) with SWCNTs to manufacture an SWCNTs/DA-CMC composite fiber-shaped strain sensor by wet spinning [169]. DA-CMC was chosen as the framework because it forms a robust framework through secondary bonding (anion-π, π-π, and van der Waals interactions as well as hydrogen bonding) and wraps CNTs to toughen their interfaces. They dispersed DA-CMC and SWCNTs in an aqueous solution. Upon coagulation in a non-solvent (ethanol), the DA-CMC and SWCNTs dispersion readily formed a composite fiber. The sensor had a high toughness (~76.2 MJ/m^3^). The strain to failure was ~14.8% at 90% relative humidity. Moreover, the sensor formed conductive networks that effectively support bending, strain, and compression in air or fluid media.

Nam et al. developed an MWCNTs/(3,4-ethylenedioxythiophene):poly(styrene sulfonate) (PEDOT:PSS)/natural rubber composite fiber-shaped strain sensor (Figure 5g) [165]. The sensor was fabricated by coaxial wet spinning. PEDOT:PSS acted as bridges to connect MWCNTs and improved the conductivity and linearity of the sensor. Natural rubber was used as the matrix due to its outstanding mechanical properties and broad working range (2500%). The sensor had a wide strain sensing range (1275%), high linearity (up to 1000%), and durability (2000 cycles at a strain of 200%), but low sensitivity (gauge factor of 3.85). The sensor could be sewn onto fabric and detect various human motions.

Li et al. developed an MWCNTs/poly(styrene-b-ethylene-ran-butylene-b-styrene) (SEBS) composite fiber-shaped strain sensor by wet spinning (Figure 5h) [166,170]. They chose THF as a solvent and used SEBS as a matrix due to its excellent elastic properties and strong π-π interaction between SEBS and MWCNTs. The morphology and mechanical, electrical, and electromechanical properties of their sensor were examined as a function of the content and aspect ratio of MWCNTs. The tensile strength and elongation at the break of their sensor decreased with increasing MWCNT content, while the electrical conductivity and strain sensing range increased with increasing MWCNT content. When the content of MWCNTs was constant, the low aspect ratio of MWCNT achieved the highest tensile strength and elongation at break, while the high aspect ratio showed the highest electrical conductivity, stretchability, and gauge factor. The sensor had a wide strain range of 0–506%, a gauge factor of 58.2 at 0–275% strain and 197.9 at 275–506% strain, and reliable durability.

Liu developed an MWCNTs/biodegradable polyurethane (BPU) composite fiber-shaped strain sensor via wet spinning [23]. The choice of BPU as the matrix material minimizes environmental pollution at the end of the sensor’s life. They examined the strain-sensing performance of their sensor by changing the MWCNTs’ weight ratio. The sensor with 12 wt% MWCNTs exhibited a wide strain sensing range (250%), high sensitivity (gauge factors of 15 at 100% strain and 2468 at 250% strain), and durability (3000 cycles at a strain of 50%). Moreover, this sensor can be integrated into the fabric and is capable of detecting various human motions, including eye blinking and bending of fingers, wrists, elbows, and knees.

Nevertheless, CNTs/polymer composites have various limitations as strain sensors. First, the gauge factor is low due to the high initial resistance [165]. Second, they are potentially vulnerable to repeated mechanical loads, which may worsen repeatability and linearity. Repeated mechanical loads may relocate CNTs or form cracks in the polymer matrix, which would aggravate the percolation network [171,172] Finally, the manufacturing of CNTs/polymer composites strain sensor has low reproducibility because it is difficult to homogeneously disperse CNTs in polymer due to the strong van der Waals interactions among [173,174].

### 4.2. CNTF-Based Strain Sensors

Using pure CNTFs as piezoresistive material instead of CNTs/polymer composite fiber can overcome the limitations of CNTs/polymer composite fiber-shaped strain sensors. First, CNTFs have a high gauge factor due to the low initial resistance [175]. Second, since CNTFs are purely made of aligned CNTs, the percolation network of CNTs is robustly preserved after repeated mechanical loads. Thus, CNTF-based strain sensors have higher repeatability and [176,177]. Finally, the manufacturing of CNTF-based strain sensors is more reproducible since it does not require dispersing CNTs in the polymer. Table 3 summarizes representative reports on CNTF-based strain sensors.

**Figure 6:**
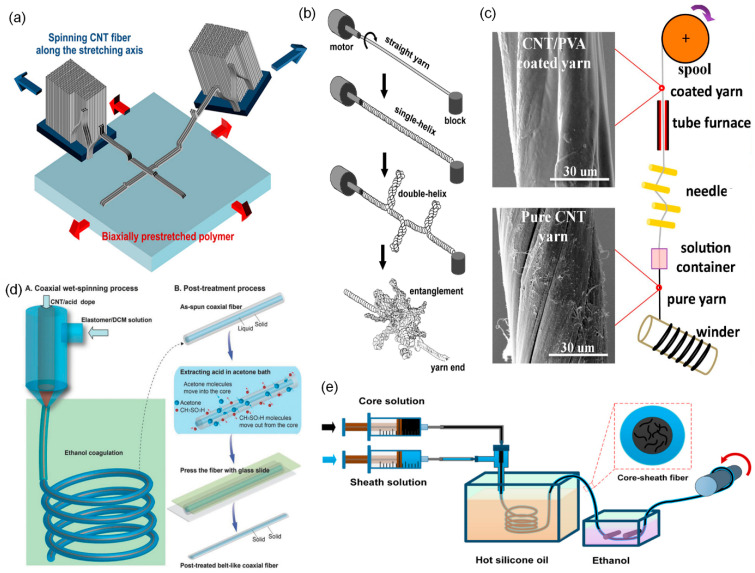
Schematic illustrations of the fabrication process of CNTF-based strain sensor. (**a**) CNTF attached directly to Ecoflex in biaxial (reprinted with permission from the study by Ryu et al. [178]), (**b**) entangled CNTF created by overtwisting a straight CNTF (reprinted with permission from the study by Li et al. [180]), (**c**) coating PVA on pure CNTF surface (reprinted with permission from the study by Li et al. [182]), (**d**) CNTF wrapped by TPE (reprinted with permission from the study by Zhou et al. [183]), and (**e**) CNTF wrapped by silicone (reprinted with permission from the study by Tang et al. [184]).

Ryu et al. reported an extremely stretchable and wearable strain sensor using dry-spun CNTFs (Figure 6a) [178]. The researchers directly affixed these CNTFs to a flexible substrate called Ecoflex, a highly elastic silicone material capable of withstanding strains exceeding 900%. Prior to attachment, they prestrained the substrate by 100% to enhance the strain sensor’s stretchability. In addition, they oriented CNTFs biaxially so that the sensor could detect strain along each axis. Their sensor could be stretched by over 900% and exhibited sensitivity with a gauge factor of 47 in the strain range of 200–440% and high durability (10,000 cycles at a strain of 300%).

Shang et al. reported an elastic CNTF strain sensor that has a helical structure by twisting SWCNT films [179]. This sensor demonstrated the ability to stretch by 25% over 1000 cycles. Similarly, Li et al. developed an overtwisted CNTF strain sensor by twisting SWCNT films (Figure 6b) [180]. Through this overtwisting process, they achieved a remarkable stretchability of 800%. However, the formation of random entanglement within the sensor led to local fluctuations in resistance during stretching and releasing. Consequently, their sensor exhibited low linearity and sensitivity (with a gauge factor of 0.12 under 500% strain). They also reported a one-meter-long SWCNT strain sensor with tunable diameter and electrical conductivity using the same technique [181].

**Table 3:** Summary of the sensing properties of CNTF-based strain sensors.

Year	Structural	Stretchability [%]	Gauge Factor	Linear Region	Durability	Ref
2013	Twisted CNTF	25	0.1 (at 25% strain)		1000 (at 25% strain)	[179]
2013	Overtwisted CNTF	800	0.1 (at 500% strain)		400 (at 500% strain)	[180]
2015	CNTF embedded in Ecoflex	900	0.5 (under 440% strain)		10,000 (at 300% strain)	[178]
			54 (within 440–900% strain)			
			[0.56 (under 200% strain),			
			47 (within 200–440% strain)] *			
2016	CNTF embedded in PDMS	15	100,000 (at 15% strain)		5000 (at 12% strain)	[175]
2018	PVA coating on CNTF	14	2.3 (at 12% strain)		20 (at 5% strain)	[182]
2018	CNTF wrapped by TPE	250	425 (within 20–100% strain)	20–100%	3250 (within 20–100% strain)	[178]
2018	CNTF wrapped by silicone	330	18,181 (at 330% strain)		10,000 (at 100% strain)	[184]
			[1378 (at 330 strain)] *			
2021	Epoxy coating on CNTF	11	8.65 (at 2% strain)	Up to 2%	20 (at 2% strain)	[181]

* These values are suspected to be miscalculated.

Zhou et al. fabricated an ultra-sensitive CNTF strain sensor inspired by human joints [175]. In their design, the CNTF served as the bone, while an elastic PDMS acted as the skin. The CNTF was produced via wet spinning, utilizing methanesulfonic acid as a solvent. Due to the inherent self-recovery ability of PDMS, this sensor was able to recover its percolation network after moderate disconnection of the percolation network. The sensor exhibited good durability (5000 cycles at a strain of 12%) and was extremely sensitive (a gauge factor of 10,000 at 15% strain).

The performance of CNTF-based strain sensors is often limited by the weak van der Waals force within the CNTF. To overcome this limitation, Li et al. coated CNTF with PVA to make a core-sheath structure (Figure 6c) [182]. PVA prevented the slippage of CNT bundles in CNTF and improved the load transfer. Compared with the pure aerogel-spun CNTF, the CNTF coated with PVA exhibited improved mechanical properties (tensile strength by 71.8%, Young’s modulus by 157.3%), resulting in a 44% increase in gauge factor (from 1.64 to 2.36 at 5% strain). Ma et al. also coated CNTF with epoxy which shows strong interfacial adhesion with MWCNTs [185]. The CNTF coated with epoxy had an improved tensile strength (145%) compared to pure direct-spun CNTF. Their sensor had moderate stretchability (11%) and sensitivity (gauge factor 8.65 at 2% strain).

To fabricate a core–sheath structure, coaxial wet spinning was also utilized. Zhou et al. developed a CNTF strain sensor wrapped by thermoplastic elastomer (TPE) using coaxial wet spinning (Figure 6d) [183]. They chose TPE as the sheath because it is an electrically insulating elastomer and prevents short-circuiting of the CNTF strain sensor. The spun fiber containing SWCNT/acid dope in the core was post-treated in acetone to remove acid residue and was pressed to densify the SWCNT core. The sensor attained high sensitivity (a gauge factor of 425 at 100% strain), high stretchability (250%), and high linearity. Tang et al. used coaxial wet spinning to fabricate a CNTF strain sensor with silicone elastomer as a sheath (Figure 6e) [184]. Silicone elastomer effectively reduced the risk of short-circuiting. The sensor exhibited a low percolation threshold (0.74 vol %), high stretchability (330%), sensitivity (a gauge factor of 1378 at 330% strain), durability (10,000 cycles at a strain of 100%), and good washability. It possessed excellent bending insensitivity and slight torsion sensitivity. They also wove the sensor onto a glove [186]. The glove strain sensor could monitor the degree of bending.

## 5. CNT-Incorporated Nanofibers for Tissue Engineering

### 5.1. CNT Nanofibers for Tissue Engineering Scaffolds

Tissue engineering is a field of scientific exploration focused on the intricate interplay of cells, signaling molecules, and scaffolds in the pursuit of regenerating, enhancing, or substituting biological tissues. Several essential criteria must be met for a material to qualify for use in tissue engineering. The successful formation of regenerated tissue is profoundly influenced by the biomaterial’s biocompatibility, as well as the architecture, composition, and three-dimensional environment of the scaffold. The porosity and distribution of pore sizes within the material significantly affect the interactions between biomaterials and host cells. Moreover, the mechanical strength of the scaffold material should align with that of the targeted tissue. In addition, the in vivo biodegradation rate of the scaffold material must be in sync with the tissue regeneration rate, and any resulting degradation byproducts must be non-harmful to the host. Consequently, it is imperative to consider how scaffolds modulate cellular interactions, as these interactions play a pivotal role in the generation of functional tissues.

CNTs have been studied extensively for their potential use in tissue engineering materials due to their unique physicochemical properties such as thermal properties, mechanical resistance, and high electrical conductivity. Also, CNTs are known to introduce extracellular matrix (ECM)-like electroconductive properties in scaffolds and stimulate certain types of cellular behavior. They can be integrated into scaffolds to provide electrical cues to cells, promoting their growth, differentiation, and tissue regeneration. Electrical stimulation has been shown to enhance the formation of various tissues, including nerves, muscles, and bones [187]. Due to such characteristic properties of CNTs, they are ideal candidates for the development of biomedical tissue engineering scaffolds.

**Figure 7:**
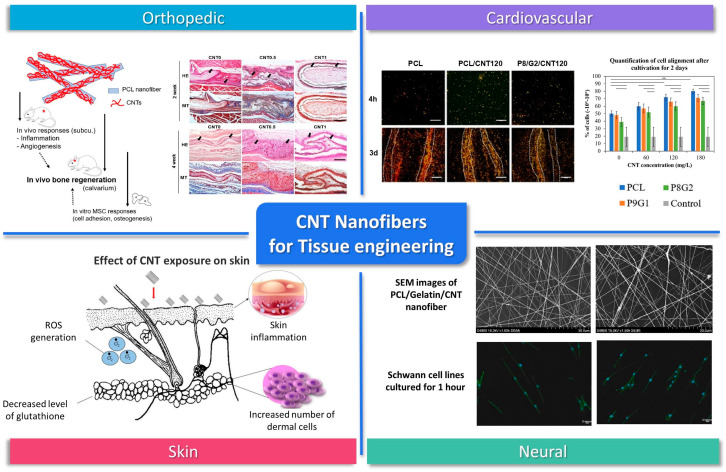
CNT nanofibers for tissue engineering (error bars represent the standard deviation, ** represents *p* < 0.01, reprinted with permission from the study by Patel et al. [190], reprinted with permission from the study by Jiang et al. [191], reprinted with permission from the study by Mohanta et al. [192], reprinted with permission from the study by Tsai et al. [193]).

Nanofibers are expected to be a suitable form of scaffolds for tissue engineering because of their nanotopological three-dimensional structure which offers a large surface area to volume ratio and porosity similar to ECM [188]. Nanofibers-incorporated therapeutics have been widely used as excellent substrates for the regulation of diverse cell behaviors such as adhesion, growth, proliferation, and differentiation [189]. Recently, the use of CNT composite nanofibers has been reported in various tissue regenerations, including bone, cartilage, neural, skin, and cardiovascular tissue (Figure 7). The incorporation of CNTs into the nanofiber structure resulted in a notable reduction of inflammatory markers, an elevation in scaffold conductivity, and a facilitation of angiogenic responses, collectively contributing to the advancement of the tissue healing process. Additionally, CNT-incorporated nanofibers provide a vital foothold for regrowing cells and reinforce mechanical integrity, thereby promising enhanced efficacy in tissue repair and regeneration applications [190].

### 5.2. CNT-Nanofiber-Based Tissue Engineering

Among various tissue regenerations, orthopedic regeneration is one of the most studied areas as the durability and flexibility of CNTs are promising for the orthopedic field. Also, it has been reported that CNTs have an affinity to bone tissue.

Patel, K. D. et al. prepared CNT-coated nanofibers for bone regeneration by reacting alkaline-modified PCL nanofibers with acid-treated multi-walled CNTs [190]. In vivo results showed that CNTs could reduce the inflammatory signals and promote angiogenesis. In vitro adhesion responses using rat MSCs were also monitored, and the number of attached cells in the CNT-coated group was noticeably high. Their results demonstrate that the incorporation of nanofibers with CNTs is a promising way of accelerating the bone tissue regeneration process. Hajrezaei, Sana et al. developed an electroconductive nanofiber scaffold with an improved piezoelectric response based on poly-L-lactic acid/polyaniline/CNT [194]. The physical and chemical properties of nanofiber were tested, and the results showed enhanced electrical conductivity and electrochemical behavior by CNT. MTT assay and live/dead staining results revealed over 85% viability for, 6 days after being cultured on CNT nanofiber. SEM images revealed strong adhesion and spreading of cells. The quantitative real-time PCR results of hBMMSCs cultured on nanofibers containing CNT showed the highest level of osteogenic differentiation. Their results demonstrate that the scaffold has great potential as an engineering substrate for bone tissue engineering.

Cartilage, unlike bones that can heal itself, lacks the ability to recover. Therefore, the achievement of the cartilage regeneration field is still insufficient. The electrical conductivity of CNTs and the three-dimensional structure of nanofiber similar to the ECM could provide an environment suitable for cartilage regeneration. Karbasi, S., and Alizadeh, Z. M. prepared Poly(3-hydroxybutyrate) (PHB)/chitosan/MWCNT nanofibers. The addition of MWCNT to PHB/chitosan resulted in better mechanical and structural properties for cartilage tissue engineering [195]. The mechanical properties results showed that the strength of the composite material with different proportions of MWCNT was about 4–10 MPa, while the strength of PHB/chitosan was 3 MPa. The water contact angle test showed that the hydrophilicity was proportional to the amount of MWCNTs. The results of this study show that the addition of CNT can produce optimal scaffolds for cartilage regeneration. Zadehnajar, P. et al. have reported that electrospun PCL-gelatin/MWCNTs nanofibers have better mechanical properties compared to PCL-gelatin nanofibers [196]. The presence of MWCNT within the scaffold slowed the degradation rate and improved the stability of the nanofiber scaffold. Furthermore, in vitro studies have confirmed that 1 wt% of MWCNT does not adversely affect the cytotoxicity or viability of chondrocytes. Their results indicate that the scaffold can provide mechanical support and is suitable for the regeneration of long-term healing tissues such as cartilage.

In neural tissue, the overall electroconductivity of the ECM is essential because the neural network between neurons with neighboring cells is a critical factor. The addition of CNTs can introduce electroconductivity in nanofiber scaffolds, facilitate neural cell growth, and guide axonal extension.

CNT-based scaffolds have been used to bridge nerve gaps and promote nerve regeneration in animal models, demonstrating their potential for repairing damaged nerves. CNT nanofibers have shown promise in nerve regeneration applications. Tsai, S.-W. et al. confirmed Schwann cells cultured on Aligned Carbon Nanotube/Polycaprolactone/Gelatin nanofibers show high cell proliferation levels with aligned typical bipolar morphologies [193]. Su, W.-T. and Y.-A. Shih fabricated PCL nanofibers with CNTs by electrospinning and promoted the differentiation of PC12 cells to neurons by electrical stimulation [197]. Cell activity and Axon formation indicate that PC12 cells can grow in samples. In addition, the significant increase of gene expressions of GAP43 and MAP1b suggests CNTs as a potential use for nerve regeneration. Lewitus et al. suggested CNT/Agarose fibers as a scaffold for neural tissue engineering. The hybrid materials are created by dispersing carbon nanotubes within an agarose matrix. The resulting fibers have a promising combination of mechanical strength, biocompatibility, and electrical conductivity for use in neural tissue engineering applications. In an in vitro test, the fibers were found to promote attachment and growth of primary brain cells. The in vivo evaluation with rats showed the presence of activated microglia and astrocytes near the implant sites [198]. Nazeri et al. used PLGA(poly(lactic-co-glycolic acid))/CNT nanofiber as a nerve conduit with multichannel structures for improving sciatic nerve regeneration in rats. The study involved bridging a 10 mm defect in rats using the conduit and measuring nerve regeneration. They suggested that the use of multichannel structures in nerve conduits, specifically with a PLGA/CNT nanofiber design, holds promise for improving nerve regeneration [199].

Skin tissue engineering is one of the most studied areas, but few studies have been reported using CNT composite nanofibers. By incorporating CNTs, the mechanical and electrical properties of the scaffold can be improved. Ince Yardimci, A. et al. cultured keratinocytes on fabricated CNT Incorporated Polyacrylonitrile/Polypyrrole nanofibers for 7 days [200]. Their results show that cells are well attached and proliferated in nanofiber with or without CNTs suggesting that CNT does not affect the biocompatibility of PAN/PPY nanofibers.

In cardiac tissue, as an electroactive tissue, cells are connected to each other via excitation-contraction coupling. CNT can provide electric stimulation and mechanical force for cardiac cell adhesion, organization, and cell-cell coupling for the contraction of cells. Shokraei, Nasim et al. fabricated electroconductive nanofibrous patches by electrospray of MWCNTs on polyurethane nanofibers [201]. The electrical conductivity, tensile strength, Young’s modulus, and hydrophilicity of CNT/PU nanofiber were enhanced after adding CNTs. In vitro results showed that the presence of MWCNT within the nanofiber improved the viability and proliferation of cardiomyocytes and promoted their electrophysiological ability. Their results demonstrate that CNT has potential application in cardiac tissue engineering by improving interactions between the scaffold and cardiomyoblasts. Mombini, Shabnam et al. developed electrically conductive nanofiber scaffolds based on polyvinyl alcohol (PVA), chitosan (CS), and 1% of CNT [202]. Mechanical test (elastic modulus: 130 ± 3.605 MPa), electrical conductivity (3.4 × 10^−6^ S/Cm), water uptake, cell adhesion, and cell viability (>80%) results indicated that CNTs significantly enhance modulus, conductivity, chemical stability, and adherence of MSCs to scaffolds. The real-time qPCR results showed upregulation of the cardiac marker of cardiomyocyte differentiation in the PVA-CS-CNT1 scaffold, demonstrating it has great potential as an engineering substrate for cardiac differentiation.

Furthermore, CNT nanofibers are suitable candidates for vascular tissue engineering due to their anisotropic fibrous structure and conductivity. Jiang, Chen et al. have reported that the inclusion of SWCNTs in PCL/gelatin nanofiber can enhance the elongation and alignment of ECs on fiber [191]. The scaffold shows a similar structure and mechanical properties to the native vessels. in vitro studies have confirmed that ECs cultured in the lumen of the scaffold proliferated and exhibited an alignment morphology, indicating polymer/CNT composite nanofibers are excellent candidates for constructing cardiovascular tissue engineering.

Several approaches for creating continuous fibers of CNT have been demonstrated. Among them, wet spinning allows the effective integration of useful molecules within the CNT fibers. In this method, CNTs in the spinning solution should dispersed using surfactants to overcome the attraction of van der Waals. The most common surfactants are lithium dodecyl sulfate (LDS), sodium dodecyl sulfate (SDS), sodium dodecyl benzene sulfonate (SDBS), and triton X-100. Several approaches for using biopolymers such as hyaluronic acid (HA), chitosan, and DNA as a surfactant and ion-conducting binder in the CNT-incorporated fiber-making system have been applied.

Razal et al. have demonstrated novel approaches for spinning fibers from CNTs using HA without the use of polymer binding agents in the coagulation system [203]. The injection of a spinning solution into the coagulation medium containing calcium chloride provided a gel-fiber structure because calcium bridges are formed between D-glucuronic acid residues in adjacent HA chains. Formed fibers were uniformly circular and had relatively smooth surfaces. HA enhanced control of fiber composition, electrical conductivity, and cytocompatibility of CNT fiber. In vitro tests showed L929 cells adhere and proliferate as well in fibers using HA as they did on TCP, whereas they did not perform well in groups using Triton X-100 as a surfactant. The results indicated that the presence of the HA in fiber can support cell adhesion and growth. Zheng et al. have produced HA/MWCNT hybrid fibers by a wet spinning method using HA as a dispersion agent [204]. They investigated the effect of HA concentration, injection speed, dispersion sonication time, and MWCNT concentration on the formation and various properties of the HA/MWCNT hybrid fiber. The obtained fibers were uniformly circular and had excellent electrical conductivity, mechanical properties, and stable behavior to be used as electrode materials, intelligent materials, conductive materials, and high-performance materials.

## 6. Conclusions

CNTFs, one-dimensional macroscopic assemblies of CNTs, realize the unique properties of CNTs in the macroscopic world. Among many application areas, the biomedical application area is where CNTFs can fully exert their uniqueness and thus have advantages over other materials. In this review, we introduced three representative biomedical applications of CNTFs: biosensors, flexible strain sensors, and tissue engineering.

First, CNTFs have shown great potential as biosensors for detecting various biological analytes, including glucose, dopamine, ascorbic acid, and so on. CNTF-based biosensors exhibit excellent sensitivity, selectivity, detection range, stability, biocompatibility, and low limit of detection. These excellent performances are attributed to several factors. First, CNTFs have high electron transfer kinetics which allow for high temporal measurement. Second, CNTFs are highly porous, so the adsorption of biomolecules is effective. Third, CNTFs exhibit high resistance to fouling caused by neurotransmitters and little degradation of performance in long-term use. Finally, CNTFs are flexible and soft, so they match soft tissues better than hard electrodes. These metrics can be enhanced by improving the electrical conductivity of CNTFs or increasing the number and strength of biomolecule adsorption. This can be achieved through methods such as acid treatment, thermal treatment, fiber coating, and mixing with functional polymers to create CNT/polymer composite fibers.

Second, utilizing their high piezoresistivity, mechanical robustness, and flexibility, CNTFs have been used as flexible strain sensors that can monitor human motion. Since CNTF-based strain sensors are flexible and mechanically robust, they can be merged onto wearable devices. So far, the research has mainly focused on improving stretchability and sensitivity. As a result, these sensors achieved high stretchability of over 100% and a high gauge factor. However, there are some challenges to overcome. These are related to the reliability of the sensors. CNTF-based strain sensors suffer from a low linearity between strain and resistance change. In addition, hysteresis is often observed.

Finally, CNTFs show promising potential for tissue engineering, but several challenges must be addressed to ensure their safety and effectiveness in clinical settings. One major challenge is achieving biocompatibility, where CNTs must not trigger adverse immune responses, inflammation, or negative reactions in the body. Additionally, the toxicity of CNTs is a concern, as some studies indicate possible harm to cells and tissues, leading to long-term health issues and even cancer. Lack of standardization in CNT production poses difficulties in maintaining consistent quality and purity, which are crucial for biomedical uses. Moreover, the high cost of manufacturing CNTs makes them less accessible for widespread use in medical devices and biomaterials. Overcoming these challenges is essential to harness the full potential of carbon nanotubes for biomedical applications.

CNTFs offer immense potential for biomedical applications due to their unique properties, including high surface area, electrical conductivity, and mechanical strength. However, common challenges exist for all these biomedical applications. Currently, although some companies are producing CNTFs, CNTFs are very expensive. Reduction of cost is imperative. The largest portion of the prediction cost is the price of CNTs. Using the wet spinning method, only high-grade SWCNTs or DWCNTs can be spun into CNTFs. Hence, research on the economical synthesis of high-grade SWCNTs and DWCNTs is required. The direct spinning method must overcome the limited productivity. Another important issue is biocompatibility. Although CNTFs have been reported to be non-cytotoxic, verification of long-term stability with respect to CNT leakage is necessary for use as an implantable biosensor. Visionary breakthroughs in CNTF technology involve enhancing biocompatibility through surface modification, tailoring properties for specific applications, and developing biodegradable materials through collaborative efforts between researchers and industry. Overcoming these challenges could pave the way for CNTFs to revolutionize biomedical fields by enabling safer, more effective bio-sensing and tissue engineering systems for biomedical applications.

## Figures and Tables

**Table 1:** Applications of CNTF biosensors. PMMA: polymethylmethacrylate, PLA: polylactic acid, PEI: polyethylenimine, ZnO: zinc oxide, PAN: polyacrylonitrile.

Analyte	Fiber Type	Spinning Method	Sensitivity	Detection Limit	Ref.
Glucose	CNT	Wet spinning			[76]
	CNT	Forest spinning	5.6 nA μM^−1^	50 μM	[77]
	CNT	Direct spinning	7.2 nA mM^−1^	2 mM	[71]
	CNT	Direct spinning	2.7 nA mM^−1^	25 μM	[78]
	CNT	Wet spinning		0.5 μM	[79]
	PMMA/CNT	Electrospinning	3.7048 nA mM^−1^	1 μM	[80]
	PLA/CNT	Solution blow spinning	358 nA mM^−1^	0.08 mM	[81]
	CNT	Direct spinning	4.867 nA mM^−1^		[82]
	CNT	Wet spinning	3.025 mA (cm^2^ mM)^−1^	1.4 μM	[83]
	CNT	Wet spinning	813 mA (cm^2^ mM)^−1^	0.59 μM	[84]
Dopamine	CNT	Wet spinning			[85]
	CNT	Wet spinning			[86]
	CNT	Forest spinning	0.28 mV nM^−1^	5 nM	[87]
	CNT	Forest spinning		13.4 ± 1.7 nM	[88]
	CNT	Forest spinning		10 ± 0.8 nM	[54]
	PEI/CNT	Wet spinning		5 nM	[74]
	PEI/CNT, CNT	Wet spinning, Forest spinning			[89]
	CNT	Forest spinning		4.6 ± 0.9 nM	[90]
Ascorbic acid	CNT	Forest spinning			[91]
	CNT			1.32 μM	[92]
Oxygen, pH	CNT	Forest spinning			[93]
Malaria biomarker(PfHRP2)	ZnO/CNT	Electrospinning	8.29 kΩg^−1^ mL	0.97 fg mL^−1^	[94]
Catechol	PAN/CNT	Electrospinning	118 mA M^−1^	0.9 μM	[95]

**Table 2:** Summary of the sensing properties of CNTs/polymer composite fiber-shaped strain sensors.

Year	Materials	Spinning Method	Stretchability [%]	Gauge Factor	Linear Region	Durability	Ref.
2015	MWCNTs/cellulose	Wet	14.3	18 (at 14.3% strain)			[159]
2016	MWCNTs/PC	Melt	9	16 (at 5% strain)			[160]
2018	MWCNTs/SBS	Wet	260	20,000 (at 14.3% strain)	Up to 1%		[161]
				175 (at 50% strain) *			
2018	MWCNTs/TPU	Wet	320	22 (under 160% strain)	0–160%	9700	[162]
				97 (within 160–320% strain)	160–320%	(at 100% strain)	
2019	MWCNTs/TPU	Wet	117	71 (at 35% strain)			[167]
				5200 (at 35% strain) *			
2019	MWCNTs/TPU	Wet	100	27 (at 100% strain)			[168]
				2800 (at 100% strain) *			
2020	MWCNTs/Ag NW/TPU	Wet	254	49 (at 254% strain)			[163]
2021	MWCNTs/PEDOT:PSS/ natural rubber	Wet	1000	2 (at 100% strain) 3.8 (at 1000% strain) *	Up to 1000%	2000 (at 200% strain)	[165]
2022	MWCNTs/SEBS	Wet	506	58.2 (under 275% strain)	0–275%	2500	[166]
				197.9 (within 275–506% strain)	275–506%	(at 20% strain)	
2022	MWCNTs/biodegradable PU	Wet	250	100 (at 200% strain)		3000	[23]
				15 (at 100% strain),		(at 50% strain)	
				2468 (at 250% strain) *			

* These values are suspected to be miscalculated.

## Data Availability

The datasets generated during and/or analyzed during the current study are available from the corresponding author upon reasonable request.
